# Molecular Signatures in the Prevention of Radiation Damage by the Synergistic Effect of N-Acetyl Cysteine and Qingre Liyan Decoction, a Traditional Chinese Medicine, Using a 3-Dimensional Cell Culture Model of Oral Mucositis

**DOI:** 10.1155/2015/425760

**Published:** 2015-01-29

**Authors:** Maria P. Lambros, Lavanya Kondapalli, Cyrus Parsa, Hari Chandana Mulamalla, Robert Orlando, Doreen Pon, Ying Huang, Moses S. S. Chow

**Affiliations:** ^1^College of Pharmacy, Western University of Health Sciences, Pomona, CA 91766, USA; ^2^College of Osteopathic Medicine, Western University of Health Sciences, Pomona, CA 91766, USA; ^3^Center for Advancement of Drug Research and Evaluation, Western University of Health Sciences, Pomona, CA 91766, USA

## Abstract

Qingre Liyan decoction (QYD), a Traditional Chinese medicine, and N-acetyl cysteine (NAC) have been used to prevent radiation induced mucositis. This work evaluates the protective mechanisms of QYD, NAC, and their combination (NAC-QYD) at the cellular and transcriptional level. A validated organotypic model of oral mucosal consisting of a three-dimensional (3D) cell tissue-culture of primary human keratinocytes exposed to X-ray irradiation was used. Six hours after the irradiation, the tissues were evaluated by hematoxylin and eosin (H and E) and a TUNEL assay to assess histopathology and apoptosis, respectively. Total RNA was extracted and used for microarray gene expression profiling. The tissue-cultures treated with NAC-QYD preserved their integrity and showed no apoptosis. Microarray results revealed that the NAC-QYD caused the upregulation of genes encoding metallothioneins, *HMOX1*, and other components of the Nrf2 pathway, which protects against oxidative stress. DNA repair genes (*XCP*, *GADD45G*, *RAD9*, and *XRCC1*), protective genes (*EGFR* and *PPARD*), and genes of the NF*κ*B pathway were upregulated. Finally, tissue-cultures treated prophylactically with NAC-QYD showed significant downregulation of apoptosis, cytokines and chemokines genes, and constrained damage-associated molecular patterns (DAMPs). NAC-QYD treatment involves the protective effect of Nrf2, NF*κ*B, and DNA repair factors.

## 1. Introduction

Mucositis is a debilitating disease that causes ulcers in the mouths or intestines of cancer patients who are treated with radiation or chemotherapy, resulting in pain and difficulty in eating and therefore contributing to their morbidity. In severe cases of mucositis, the therapeutic radiation regimen has to be modified or stopped, preventing the cancer patient from receiving the required therapy.

Mucositis develops over five phases [1]. The first is the initiation phase. Immediately after radiation or chemotherapy, reactive oxygen species form, which cause mucosal damage to the mouths or intestines of these patients. The initiation phase is considered to be a critical stage in the development of mucositis; by preventing this stage, mucositis-related injuries may be minimized. Thus, effective prevention of mucositis must happen at the initiation stage. The second is the signaling phase, which is characterized by message generation and the upregulation of cytokines and other factors. The third is the amplification phase, which is characterized by the increased production of cytokines that cause ulceration. The fourth is the bacterial colonization of ulcers. Mucositis clinically manifests at this stage. The fifth is the healing phase, during which the integrity of the mucosal layer is restored [2–4].

There are few prophylactic or therapeutic treatments for mucositis. Among the available treatments, N-acetyl cysteine (NAC) has been evaluated in a randomized, double-blind placebo controlled study of 110 patients with head and neck cancer who received daily radiation therapy. At the highest cumulative radiation therapy dose, the incidence of severe oral mucositis (grade 3 or 4) was significantly lower among patients receiving NAC [2, 5, 6]. Another therapy, Qingre Liyan decoction (QYD), is a traditional Chinese medicine decocted from a mixture of twelve different plant products: Flos Lonicerae (*Lonicera japonica*) 15 g, Belamcandae Rhizoma (*Belamcanda chinensis*) 15 g, Lasiosphaera seu calvatia (*Lasiosphaera fenzlii* Reich,* Calvatia gigantea*) 9 g, Astragali Radix (*Astragalus membranaceus*) 30 g, Glehniae Radix (*Glehnia littoralis*) 30 g, Ophiopogonis Radix (*Ophiopogon japonicus*) 30 g, Trichosanthes Radix (*Trichosanthes kirilowii*) 15 g, Scrophulariae Radix (*Scrophularia ningpoensis*) 15* *g,* Ligusticum wallichii *Rhizoma (*Ligusticum chuanxiong*) 15 g, Agrimoniae Herba (*Agrimonia pilosa*) 20 g, Imperatae Rhizoma (*Imperata cylindrica*) 9 g, and Glycyrrhizae Radix (*Glycyrrhiza uralensis*) 10 g. In a randomized trial, QYD significantly reduced the incidence of severe mucositis in patients receiving radiation therapy to the head and neck region, compared to the control group who were administered Dobell's solution [7].

Models of oral mucositis include monolayer cell cultures of human keratinocytes (2D), three-dimensional (3D) human cell tissue-culture of oral epithelia, biopsies, and in vitro reconstructed skin [8–11]. The 3D cell cultures are more realistic models compared to the 2D cell cultures because cells live in three dimensions. A 3D model of human oral epithelia has been established and the effect of different doses of radiation has been evaluated using histology and transcriptional studies [8].

In this study, we used a validated organotypic model of oral mucosal tissues consisting of a 3D cell tissue-culture of primary human keratinocytes, and we evaluated the prophylactic effects of NAC, QYD, and NAC-QYD by studying the tissue histology, apoptosis, and gene expression. We found that tissues treated with NAC-QYD preserved their integrity and showed no apoptosis. Microarrays showed that NAC-QYD-treated tissue had significantly upregulated* metallothioneins*,* HMOX1*, and other genes of the Nrf2 pathway, which protect cells against oxidative stress; DNA repair genes were also significantly upregulated, as well as* EGFR*,* PPARD*, and genes encoding the NF*κ*B pathway. Furthermore, NAC-QYD-treated tissues showed significantly downregulated cytokines and chemokines as well as constrained DAMPs.

## 2. Materials and Methods

### 2.1. Three-Dimensional (3D) Tissues

This study utilized 3D human cell culture tissues (EpiOral MatTek Corporation, Ashland, MA), which have been validated as a human tissue substitute of oral mucosal. This is an organotypic model which consisted of primary human buccal keratinocytes grown in Millipore Millicell tissue-culture plate inserts using serum-free media at 37°C with 5% CO_2_. The attained 3D cultures were highly differentiated and morphologically similar to human buccal epithelia with an organized basal layer and multiple apical noncornified layers. The 3D EpiOral tissue and media (containing specially prepared phenol red, 5 *μ*g/mL gentamicin, and 0.25 *μ*g/mL amphotericin B) were purchased from MatTek Corporation (Ashland, MA). For this experiment, the apical surface of the 3D tissues was exposed to 100 *μ*L of one of the following mixtures for 2 h at 37°C: (a) 1 mM NAC, (b) 5 mg/mL QYD, or (c) an NAC-QYD mixture consisting of 1 mM NAC and 4.5 mg/mL QYD. Then, the tissues were rinsed with phosphate-buffered saline (PBS) to remove the treatment materials and transferred to new plates with fresh culture medium. At least three independent 3D oral tissues were used for each treatment.

### 2.2. Irradiation

The 3D oral tissues were irradiated at the City of Hope (Duarte, CA) facility. The dose of the gamma irradiation exposure was 12 Gy. After irradiation, the tissues were incubated for 6 h at 37°C with 5% CO_2_. Subsequently, some of the tissues were used for the extraction of total RNA, and others were placed in 10% formalin for histopathological studies.

### 2.3. TUNEL Assay

A TUNEL assay with the Cell Death Detection Kit (Roche, Philadelphia, PA) was used to visualize apoptotic cells. The 3D tissue was treated with proteinase K and rinsed twice. Then, a buffer containing a labeled nucleotide mix and TdT enzyme was added to the tissue, and the samples were incubated in a humidified chamber at 37°C for 1 h. TUNEL staining was observed using a fluorescence microscope (Nikon Eclipse, Nikon Instruments, Inc., Melville, NY).

### 2.4. Traditional Chinese Medicine Qingre Liyan (QYD)

The Traditional Chinese medicine, Qingre Liyan decoction, was provided by Dr. Moses S. S. Chow. It was prepared fresh from powder just before the experiments in serum-free media specially formulated by MatTek Corporation (Ashland, MA) (containing phenol red, 5 *μ*g/mL gentamicin, and 0.25 *μ*g/mL amphotericin B) and sonicated for 30 minutes. The mixture was centrifuged at 3,000 rpm for four minutes and the supernatant was used to treat the 3D tissues.

### 2.5. Histology

The 3D tissue samples were placed in 10% formalin. The tissues were then stained with hematoxylin and eosin (H and E) and evaluated microscopically to assess the prophylactic effect of the treatments on the irradiation damage.

### 2.6. Gene Expression

The RNeasy Plus Mini Kit (Qiagen, Germantown, MD) was used to extract total RNA from the treated and untreated 3D tissues. A DNA microarray analysis was performed using the Human Whole Genome OneArray (Phalanx Biotech, Palo Alto, CA). The quality and integrity of the RNA were determined using an Agilent 2100 Bioanalyzer (Agilent Technologies, Palo Alto, CA) by monitoring the A260/280 absorbance. Only RNA of the highest quality was used for analysis (RIN > 7.0 and A260/280 absorbance ratio > 1.8).

RNA was converted to double-stranded cDNA and amplified using an in vitro transcription reaction that included aminoallyl UTP. Then, the cDNA product was conjugated to Cy5 NHS ester (GE Healthcare Life Sciences, Pittsburg, PA). Fragmented RNA was hybridized overnight at 42°C using the HybBag mixing system with 1 × OneArray Hybridization Buffer (Phalanx Biotech, San Diego, CA) and 0.01 mg/mL sheared salmon sperm DNA (Promega, Madison, WI). The labeled target concentration was 0.025 mg/mL. After hybridization, the arrays were washed according to the OneArray protocol.

A Molecular Dynamics Axon 4100A scanner was used to capture the raw intensity signals produced by each of the microarrays. The signals were measured using GenePix Pro software and stored in GPR format. The Rosetta Resolver (Rosetta Biosoftware) was used to analyze the data from all microarrays in each experimental set. Testing was performed in triplicate by combining technical replicates and performing statistical analyses using the proprietary modeling techniques of the Rosetta Resolver. Then the data were imported into a Microsoft Excel database, with the corresponding gene names. The data were also deposited to Gene Expression Omnibus (GEO, accession number GSE62397).

## 3. Results and Discussion

### 3.1. Tissues Pretreated with NAC-QYD Are Not Affected by Radiation

**Figure 1:**
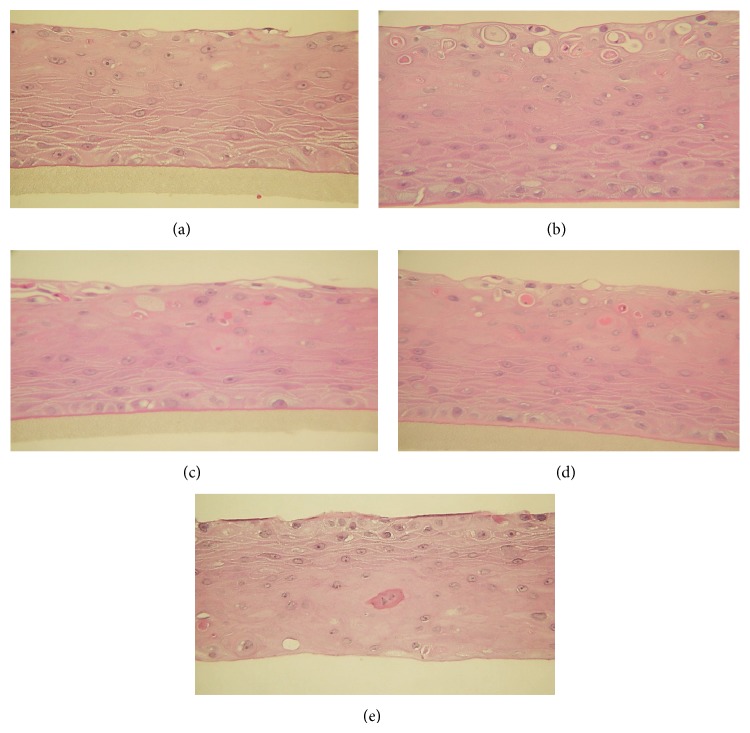
H and E staining of 3D-cultured human oral tissue. Nonirradiated, nontreated oral epithelium (negative control) consists of human oral keratinocytes and is divided into the stratum basalis (basal layer), stratum spinosum (the spindle shaped cell layer), and the stratum corneum (a); irradiated, nontreated tissue (positive control). Keratinocyte injury is noticeable at the top of the tissues, with an increased number of pyknotic cells at the bottom of the tissue (b); irradiated tissue prophylactically treated with NAC shows keratinocyte injury at the top of the tissue (c); irradiated tissue prophylactically treated with QYD shows keratinocyte injury (d); irradiated tissue prophylactically treated with NACQYD shows no injury and resembles the nonirradiated, nontreated negative control tissues (e).

**Figure 2:**
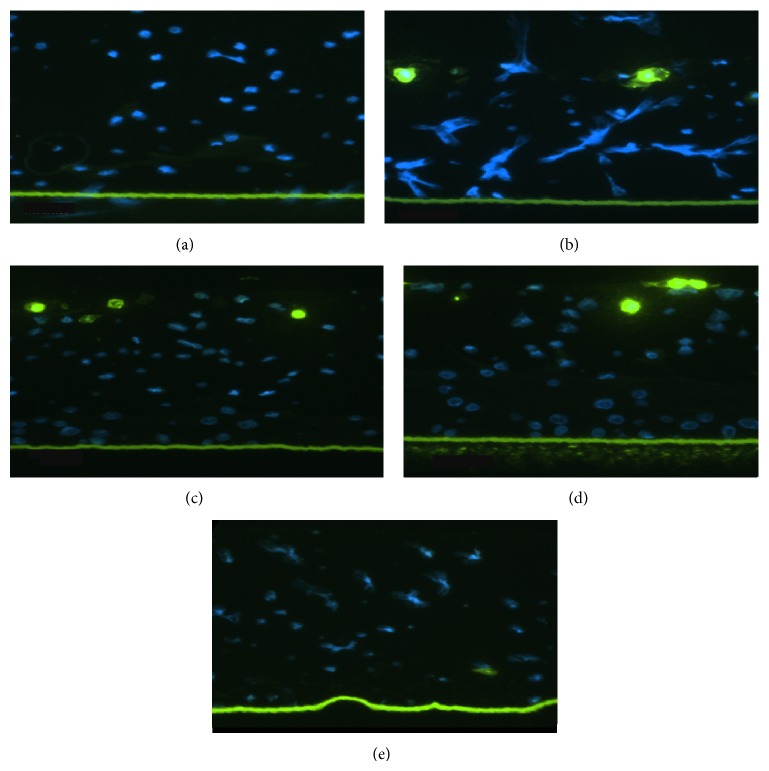
The apoptotic cells are stained in bright green. Nonirradiated, nontreated tissue (a); irradiated, nontreated tissue (b); irradiated tissue treated with NAC (c); irradiated tissue treated with QYD (d); irradiated tissue treated with NAC-QYD shows no apoptotic cells (e).

Using a 3D human cell culture model of oral keratinocytes, we studied the protective effect of three treatments on radiation damage. H and E staining of the nonirradiated tissue revealed a healthy, well-differentiated multilayer epithelium consisting of keratinocytes. In the lower part of the epithelium, the stratum basalis and stratum spinosum layers, consisting of cylindrical cells and elongated spindle cells, respectively, could be distinguished (Figure 1(a)). Untreated tissue samples irradiated with 12 Gy showed laceration and damage to the top part of the tissues (Figure 1(b)) and the presence of apoptotic cells (Figure 2(b)). Tissues pretreated with NAC before irradiation also revealed the formation of edematous and apoptotic cells (Figures 1(c) and 2(c)). The pretreatment of tissues with QYD showed fewer edematous cells (Figure 1(d)) compared with those pretreated with NAC (Figure 1(c)); however, the pretreatment with NAC-QYD resulted in healthy 3D tissue (Figure 1(e)) similar to the findings in the nonirradiated tissue.

The TUNEL assays revealed the absence of apoptotic cells in the nonirradiated (control) (Figure 2(a)) and NAC-QYD tissues (Figure 2(e)); in contrast, the irradiated, untreated tissues and the irradiated, NAC- and QYD-pretreated tissues showed the presence of apoptotic cells (Figures 2(b)–2(d)).

### 3.2. NAC-QYD Treatment Affords Protection from Radiation via Nrf2 and DNA Repair

In this experiment, the genes that encode components of the nuclear factor erythroid 2-related factor (Nrf2) pathway, such as* HMOX1*,* MT1E*, and* MT2A*, were significantly upregulated by the NAC-QYD treatment, whereas* G6PD*,* NQO2*,* TXNRD1*, and* UGT1A10* were significantly upregulated by both the QYD and the NAC-QYD treatments (Table 1). Ionizing radiation causes cells to experience oxidative stress, which the cells must counteract to maintain homeostasis. Nrf2 is a transcription factor and a key controller of cell redox homeostasis [12]. Upregulated Nrf2 helps cells to counteract oxidative stress and survive [13, 14]. Upregulation of Nrf2 protected skin cells treated with Feverfew extracts from UV oxidative damage and induced DNA repair [15].

In this study, genes encoding metallothioneins (MTs) were among those that were the most upregulated, more than 10-fold in the case of the combination NAC-QYD treatment (Table 1). Metallothioneins reduce free radical species, regulate redox and apoptotic states, and have been shown to benefit cases of drug-induced toxicity and sepsis [16, 17].* MT1* and* MT2* mRNA levels were also increased after the treatment of HEPG2 cells with sulforaphane [18].

There was also significant upregulation of the heme oxygenase-1 gene,* HMOX1* (which is also known as HO-1) in the NAC-QYD prophylactically treated tissues (Table 1). The upregulation of* HMOX1* is a hallmark of Nrf2 activation [19]. HMOX1 is a cytoprotective enzyme which inhibits the reactive oxygen species (ROS) and induces anti-inflammatory responses [20, 21]. Increased* HMOX1* expression is observed in endothelial cells and is triggered by TNFa via the NF*κ*B pathway [22]. Our data parallel this finding; the upregulation of* HMOX1* was accompanied by the increased expression of several genes that belong to the NF*κ*B pathway, as discussed later.

Another gene that encodes an antioxidant protein,* NQO2*, was upregulated by the QYD and NAC-QYD treatments (Table 1). NQO2 is a flavoprotein that catalyzes the metabolic reductive detoxification of redox cycling quinones [23]. Moreover, the glucose-6-phosphate dehydrogenase (*G6PDH*) and thioredoxin reductase 1 (*TXNRD1*) were significantly upregulated in the QYD- and NAC-QYD-treated tissues (Table 1). Both genes are transcriptionally regulated by Nrf2.* G6PDH* is of crucial importance to cells for protection from oxidative damage and is involved in the generation of NADPH which preserves the redox potential of the cell. TXNRD1 is also important for detoxification and maintaining the cellular redox potential so that the cell can respond appropriately to stresses such as inflammation [24, 25].

Tissues that received the combination NAC-QYD treatment demonstrated an upregulation of DNA repair genes such as* GADD45G*,* RAD9*,* XPC*, and* XRCCI* (Table 1). The GADD45G protein plays a protective role in cells and enhances cell survival by inducing DNA repair and arresting the cell cycle [26]. In our study,* JUND* was significantly upregulated (5-fold) by the combination treatment (Table 1). The deletion of* JUND* is known to induce oxidative stress; the corresponding protein has been shown to provide protection in age-related endothelial dysfunction [27].

### 3.3. NF*κ*B Activation in NAC-QYD-Pretreated Tissues

Several genes that belong to the NF*κ*B pathway were differentially expressed between the control and the combination NAC-QYD-treated or single agent-treated tissues.* RELA*,* RELB*,* REL*,* and NF*κ*B2* were significantly upregulated in the NAC-QYD-treated tissues (Table 1). NF*κ*B regulates many diverse cell functions related to immune, inflammatory, and apoptotic responses. NF*κ*B levels are affected by irradiation and are involved in the prevention of apoptosis, facilitate DNA repair, and contribute to cell radio-resistance [28–31].

The activation of NF*κ*B leads to the induction of inflammatory cytokines; however, NF*κ*B also induces genes, such as* TNFAIP3* (A20), which reduce the extent and duration of the inflammatory response, thereby preventing inflammation from causing further tissue damage [32]. In our study,* TNFAIP3* was upregulated in the NAC-QYD tissue but downregulated in the NAC-treated and nontreated irradiated tissues, which may explain the observation that genes encoding some cytokines and chemokines, such as CXL1, CXL6, CXL14, CXL16, CCL18, and CCL20, were downregulated in the tissues treated with NAC-QYD (Table 1). TNFAIP3 also perturbs caspase activation of TNF receptor 1 (TNFR1) which is coupled to apoptotic caspases 8 and 10 [28, 33].

In our study,* FADD*,* CASP1*,* CASP8*, and* CASP10* were significantly downregulated in NAC-QYD tissue. This finding also supports the TUNEL studies, which show no apoptosis in the NAC-QYD-treated tissues. Other genes that act in synergy with NF*κ*B are* SP1*,* STAT3*, and* CEBPB*, which were found to be upregulated in the tissues that received the NAC-QYD treatment. Cross talk among NF*κ*B, SP1, and STAT3 is important for wound healing, whereas silencing the corresponding genes has been shown to impair wound healing in keratinocytes [34].

Our study showed that the combination treatment may have enhanced the radio-protective role of NF*κ*B and concurrently controlled the NF*κ*B-mediated inflammation via the upregulation of* TNFAIP3*. The radio-protective role of NF*κ*B has previously been demonstrated in the case of irradiated intestinal epithelial cells [35, 36].

**Figure 3:**
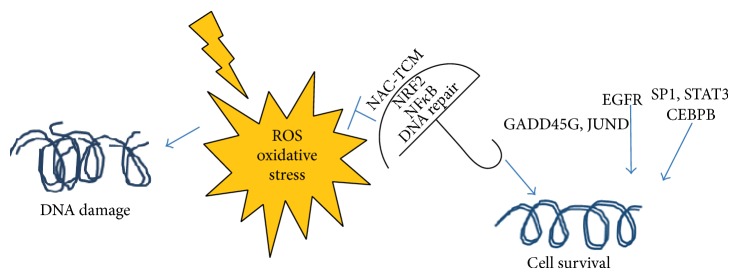
A schematic representation of the proposed molecular signatures which were elicited by the synergistic effect of NAC-QYD and prevented the radiation damage.

*EGFR* was upregulated significantly in the NAC-QYD-treated tissues and downregulated in the nontreated irradiated tissues. Signaling through EGFR has been shown to induce NF*κ*B activation and enhance cell survival, whereas blocking EGFR signaling results in the inhibition of NF*κ*B [37, 38]. A schematic representation of the protective molecular signatures discussed above is shown in Figure 3.

### 3.4. Constrained DAMPs in Tissues Pretreated with NAC-QYD

Pathogens and trauma cause damage to tissues and cells. Cells respond to this damage by releasing molecules that announce the injury to the surrounding tissues. Molecular patterns of endogenous molecules that signal tissue injury are called DAMPs. DAMPs are released by stressed cells that undergo necrosis and promote inflammatory responses. Common DAMPs include the expression of the toll-like receptors (TLRs), calcium-binding protein (S100A), the receptor of advanced glycosylation end-products such as* RAGE* (which is also known as* AGER*), and serum amyloid protein (*SAA*).

In this study,* S100A16* and* RAGE* were downregulated by the NAC-QYD treatment, whereas* S100A12*,* S100A11*, SAA1, and SAA2 were upregulated in the irradiated, nontreated control (Table 2). The upregulation of the* S100* genes after irradiation is in agreement with results from our own and others' previous studies [8, 39]. S100 proteins are markers of inflammation [40].* RAGE*, which was significantly downregulated in the QYD and NAC-QYD treatments, encodes a multiligand receptor that can initiate and perpetuate inflammatory responses and interacts with S100 proteins [41]. SAAs are considered to be markers for inflammatory disease and are potent stimulators of G-CSF, which is important in the regulation of granulocytosis. The effect of SAAs depends on TLR2. In our experiment, TLR2 was downregulated in the NAC-QYD combination-treated tissue. Blocking TLR2 has been shown to ablate the ability of SAAs to stimulate G-CSF [42].

In summary, tissues treated prophylactically with the NAC-QYD combination remained primarily unaffected by the irradiation and, in terms of histology and apoptosis, resembled the nonirradiated tissue. These findings are in contrast with the tissues treated with NAC or QYD alone which showed some irradiation damage. This prophylactic effect is due to the enhanced activity of the Nrf2 and NF*κ*B pathways, upregulation of DNA repair genes, reduced expression of chemokines and cytokines such as* CXL1*,* CXL2/CXL3*,* CXL6*,* CXL14*,* CCL18*, and* CCL20*, and constrained expression of DAMPs such as* RAGE*,* S100*, and* SAA*.

## Figures and Tables

**Table 1:** Genes pertaining to Nrf2 and NF*κ*B pathways, cytokines, and inflammatory response that are upregulated or downregulated in NAC, QYD, or NAC-QYD pretreated human 3D-cultured oral tissues after 12 Gy irradiation.

Gene symbol	Fold change compared with nonirradiated and untreated control (^*^ *P* < 0.05)				Entrez gene	Gene description
	Untreated	NAC	TCM	NAC : TCM		
*HMOX1 *	1.12	1.02	1.07	4.80^*^	3162	Heme oxygenase (decycling) 1
*MT1E *	0.76^*^	0.61^*^	0.36^*^	15.70^*^	4493	Metallothionein 1E
*MT2A *	1.22	0.79	0.74^*^	10.41^*^	4502	Metallothionein 2A
*G6PD *	1.24	1.20	1.83^*^	2.81^*^	2539	Glucose-6-phosphate dehydrogenase
*NQO2 *	1.15	1.32	1.35^*^	1.58^*^	4835	NAD(P)H Dehydrogenase, quinone 2
*TXNRD1 *	0.43^*^	0.85	3.07^*^	3.14^*^	7296	Thioredoxin reductase 1
*UGT1A10 *	1.02	1.23	2.25^*^	1.59^*^	54659	UDP Glucuronosyltransferase 1 family, polypeptide A10
*MT1IP *	1.55	1.70^*^	1.11	12.99^*^	644314	Metallothionein 1IP (pseudogene)
*MT1X *	1.82^*^	1.84^*^	1.51^*^	10.50^*^	4501	Metallothionein 1X
*GADD45G *	1.13	1.07	1.01	1.53^*^	10912	Growth arrest and DNA damage inducible gamma
*RAD9A *	0.98	1.11	1.29	2.06^*^	5883	RAD9 homolog A
*XPC *	1.18	1.47^*^	1.57^*^	1.39^*^	7508	Xeroderma pigmentosum, complementation group C
*XRCC1 *	1.01	1.50^*^	1.46^*^	1.38^*^	7515	X-ray repair complementing defective Repair in Chinese hamster cells 1
*JUND *	1.81^*^	1.87^*^	2.43^*^	5.02^*^	3727	Jun D protooncogene
*SIRT1 *	0.69^*^	0.76^*^	1.03	0.87	23411	Sirtuin (silent mating type information regulation 2 homolog) 1 (*S. cerevisiae*)
*PPARD *	0.86	0.83	0.50^*^	1.85^*^	5467	Peroxisome proliferator-activated receptor delta
*RELA *	1.27	1.04	1.54^*^	1.86^*^	5970	v-Rel reticuloendotheliosis viral oncogene homolog A (avian)
*RELB *	1.136	1.04	1.26	2.30^*^	5971	v-Rel reticuloendotheliosis viral oncogene homolog B
*NFKB2 *	1.10	1.10	1.50^*^	2.12^*^	4791	Nuclear factor of kappa light polypeptide gene enhancer in B-cells 2 (p49/p100)
*TNFA1P3 *	0.76^*^	0.68^*^	0.91	1.25^*^	7128	Tumor necrosis factor, alpha-induced protein 3
*NFKBIA *	0.83	0.96	1.01	1.25^*^	4792	Nuclear factor of kappa light polypeptide gene enhancer in B-cells inhibitor, alpha
*CXCL1 *	1.72^*^	0.54^*^	0.29^*^	0.27^*^	2919	Chemokine (C-X-C motif) ligand 1 (melanoma growth stimulating activity, alpha)
*CXCL2/CXCL3 *	2.13^*^	0.91	0.55^*^	0.54^*^	2920∣2921	Chemokine (C-X-C motif) ligand 2/chemokine (C-X-C motif) ligand 3
*CXCL6 *	1.22	1.04	0.65^*^	0.69^*^	6372	Chemokine (C-X-C motif) ligand 6 (granulocyte chemotactic protein 2)
*CXCL14 *	0.91	1.17	0.67^*^	0.51^*^	9547	Chemokine (C-X-C motif) ligand 14
*CCL18 *	0.96	1.03	0.73	0.38^*^	6362	Chemokine (C-C motif) ligand 18 (pulmonary and activation-regulated)
*CCL20 *	1.17	0.42^*^	0.42^*^	0.49^*^	6364	Chemokine (C-C motif) ligand 20
*FADD *	0.99	0.75^*^	0.97	0.70^*^	8772	Fas- (TNFRSF6-) associated via the death domain
*CASP1 *	0.78	0.54^*^	0.44^*^	0.44^*^	834	Caspase 1, apoptosis-related cysteine peptidase (interleukin-1, beta, convertase)
*CASP8 *	0.81	0.53^*^	0.58^*^	0.59^*^	841	Caspase 8, apoptosis-related cysteine peptidase
*CASP10 *	0.97	1.08	1.03	0.70^*^	843	Caspase 10, apoptosis-related cysteine peptidase
*SP1 *	1.15	1.51^*^	1.61^*^	1.82^*^	6667	Sp1 transcription factor
*STAT3 *	0.92	1.31^*^	1.22	1.32^*^	6774	Signal transducer and activator of transcription 3 (acute-phase response factor)
*CEBPB *	1.5^*^	1.03	1.25	2.22^*^	1051	CCAAT/enhancer binding protein, beta
*EGFR *	0.74^*^	0.95	1.07	1.48^*^	1956	Epidermal growth factor receptor (erythroblastic leukemia viral (v-erb-b) oncogene homolog, avian)
*IL1B *	0.96	0.78	2.93^*^	1.25^*^	3553	Interleukin-1, beta
*IL1R1 *	0.91	0.98	1.44^*^	1.54^*^	3554	Interleukin-1 receptor, type I
*TNFS10 *	0.93	0.89	0.62^*^	0.46^*^	8743	Tumor necrosis factor (ligand) superfamily, member 10
*IRZAK1BP1 *	1.01	0.86	0.68^*^	0.52^*^	134728	Interleukin-1 receptor-associated kinase 1 binding protein 1
*IRAK2 *	0.9	1.02	1.18	1.73^*^	3656	Interleukin-1 receptor-associated kinase 2
*MYD88 *	0.65^*^	0.82	0.76^*^	1.10	4615	Myeloid differentiation primary response gene (88)
*CHUK *	0.72^*^	0.74^*^	0.939	0.89	1147	Conserved helix-loop-helix ubiquitous kinase
*NFKBIE *	0.96	0.85	0.88	1.59^*^	4794	Nuclear factor of kappa light polypeptide gene enhancer in B-cells inhibitor, epsilon
*NFKBIB *	0.77^*^	1.25^*^	1.33^*^	1.08	4793	Nuclear factor of kappa light polypeptide gene enhancer in B-cells inhibitor, beta
*BCL2L10 *	1.47	1.70	1.09	0.57	10017	BCL2-like 10 (apoptosis facilitator)
*JUNB *	1.10	0.87	1.26	1.65^*^	3726	Jun B protooncogene
*CDKN1A *	1.16	1.61^*^	1.60^*^	2.42^*^	1026	Cyclin-dependent kinase 1A (p21, Cip1)

^*^P < 0.05.

**Table 2:** Genes related to damage-associated molecular patterns (DAMPs) that are upregulated or downregulated in NAC, TCM or NAC-TCM pretreated human 3D-cultured oral tissues after 12 Gy irradiation.

Gene symbol	Fold change compared with nonirradiated and untreated control (^*^ *P* < 0.05)				Entrez gene	Gene description
	Untreated	NAC	TCM	NAC-TCM		
*S100A16 *	0.94	0.98	0.88	0.76^*^	140576	S100 calcium-binding protein A16
*S100A12 *	1.47^*^	1.05	1.24	1.20	6283	S100 calcium-binding protein A12
*S100A11 *	1.29^*^	1.03	1.20	1.09	6282	S100 calcium-binding protein A11
*RAGE (AGER) *	1.10	1.18	0.77^*^	0.57^*^	177	Receptor of advanced glycosylation end-products
*SAA1 *	1.32^*^	1.59^*^	0.89	0.87	6288	Serum amyloid A1
*SAA2 *	1.20^*^	1.23^*^	1.08	1.00	6289	Serum amyloid A2
*TLR2 *	1.10	1.07	0.79	0.70	7097	Toll-like receptor 2
*IL1R2 *	0.36^*^	0.56^*^	0.53^*^	0.52^*^	7850	Interleukin-1 receptor, type II
*IL1A *	0.71^*^	0.78	1.69^*^	0.98	3552	Interleukin-1A

^*^P < 0.05.

## Abbreviations

QYD: Qingre Liyan decoction
TCM: Traditional Chinese medicine
Nrf2: Nuclear factor erythroid 2-related factor
DAMPs: Damage-associated molecular patterns
TLRs: Toll-like receptors
G6PDH: Glucose-6-phosphate dehydrogenase
HMOX1: Heme oxygenase-1
MT: Metallothionein.
